# The Pro-Resolving Lipid Mediator Maresin 1 (MaR1) Attenuates Inflammatory Signaling Pathways in Vascular Smooth Muscle and Endothelial Cells

**DOI:** 10.1371/journal.pone.0113480

**Published:** 2014-11-19

**Authors:** Anuran Chatterjee, Anjali Sharma, Mian Chen, Robert Toy, Giorgio Mottola, Michael S. Conte

**Affiliations:** Cardiovascular Research Institute (CVRI) and Department of Surgery, University of California San Francisco, San Francisco, California; University of Southampton School of Medicine, United Kingdom

## Abstract

**Objective:**

Inflammation and its resolution are central to vascular injury and repair. Maresins comprise a new family of bioactive lipid mediators synthesized from docosahexaenoic acid, an ω-3 polyunsaturated fatty acid. They have been found to exert anti-inflammatory and pro-resolving responses in macrophages, neutrophils and bronchial epithelial cells and impart beneficial actions in murine models of peritonitis and colitis. We investigated the impact of maresin-1 (MaR1) on tumor necrosis factor alpha (TNF-α) induced inflammatory responses in human vascular endothelial (EC) and smooth muscle cells (VSMC).

**Methods:**

Primary cultures of human saphenous vein EC and VSMC were employed. We tested the naturally occurring MaR1 as modulator of TNF-α effects, with examination of monocyte adhesion, oxidant stress, and intracellular inflammatory signaling pathways.

**Results:**

MaR1 attenuated TNF-α induced monocyte adhesion and reactive oxygen species (ROS) generation in both EC and VSMC, associated with down-regulated expression (cell surface) of the adhesion molecule E-selectin (in EC) and NADPH-oxidases (NOX4, NOX1, NOX2). MaR1 attenuated TNF-α induced release of pro-inflammatory mediators by EC and VSMC. MaR1 caused an attenuation of TNF-α induced NF-κB activation in both cell types associated with inhibition of I-κ Kinase (IKK) phosphorylation, IκB-α degradation and nuclear translocation of the NF- κB p65 subunit. MaR1 also caused a time-dependent increase in intracellular cyclic AMP (cAMP) in both naive and TNF-α stimulated VSMC and EC.

**Conclusions:**

MaR1 has broad anti-inflammatory actions on EC and VSMC, which may be partly mediated through up-regulation of cAMP and down-regulation of the transcription factor NF-κB. The results suggest that the pro-resolving lipid mediator MaR1 exerts homeostatic actions on vascular cells that counteract pro-inflammatory signals. These findings may have direct relevance for acute and chronic states of vascular inflammation.

## Introduction

Acute inflammatory responses are associated with vascular endothelial (EC) and smooth muscle cell (VSMC) activation and transmigration of leukocytes across blood vessels, resulting in vascular leak and edema at the site of infection or injury. Counter-regulatory mechanisms such as production of anti-inflammatory cytokines and negative feedback loops of pro-inflammatory signals blunt the inflammatory response and assist in the attainment of homeostasis. It has further become apparent that distinct bioactive mediators regulate the “resolution phase” of inflammation. Employing an unbiased lipidomics approach using liquid chromatography mass spectrometry (LC/MS-MS), novel ω3-polyunsaturated fatty acid derived lipids were discovered in mouse peritoneal inflammatory exudates, giving rise to the discovery of a new genus of “specialized pro-resolving mediators (SPM)” [1]. Docosahexaenoic acid (DHA) and eicosapentaenoic acid (EPA) present in blood and edema were found to serve as precursor ω3-PUFA for SPMs, which include protectin D1, D-series (Resolvin-D1, D2, D3, D4, D5) and E-series resolvins (Resolvin-E1, E2). Maresins (**Ma**crophage mediators in **res**olving **in**flammation) are a newly discovered class of lipid mediators synthesized by macrophages in the presence of DHA [2]. Maresin1 (MaR1) is biochemically synthesized from 14-lipoxygenation of DHA by human macrophage 12-lipoxygenase (hm12-LOX) that produces 14-hydroperoxy-docosahexaenoic acid (14-HpDHA which in turn produces an epoxide intermediate 13S, 14S-epoxide that is hydrolyzed to bioactive MaR1. The complete stereochemistry of MaR1 is established and shown to be 7R, 14S-dihydroxy- docosa-4Z,8E,10E,12Z,16Z,19Z-hexaenoic acid [3].

The profile of biological activity of SPMs is an area of considerable interest in the field of inflammation [4]. Resolvins are extensively investigated both *in vitro* and *in vivo* in models of inflammatory diseases, and were found to induce “pro-resolution” activities through cessation of neutrophil infiltration, enhancement of macrophage efferocytosis (removal of dead cells from the inflammatory milieu), and showed dose-dependent actions on attenuation of organ injury, inflammatory signaling and mortality [5]. The mechanisms through which resolvins exert their biological actions involve down-regulation of NF-κB and AP-1 activity and are thought to be mediated via G-protein coupled receptors (GPCRs) [6], [7]. Along these lines, maresins also enhance macrophage phagocytosis of apoptotic neutrophils, limit neutrophil infiltration (in a mouse model of zymosan induced peritonitis) and reduce neuropathic pain in mice by blocking transient receptor potential V1 (TRPV1) currents in dorsal root ganglion neurons [3]. In a recent study, MaR1 was shown to exert a protective effect on human bronchial epithelial cells exposed to organic dust by attenuating cytokine production and PKCα and PKCε activation [8]. In a murine model of colitis, MaR1 dose-dependently reduced colon injury, blocked expression of inflammatory mediators and reduced NF-κB activation in the colon [9]. Interestingly, it was also shown that higher levels of MaR1 were associated with an increase in M2 macrophages (associated with homeostasis of inflammation) versus M1-macrophages (pro-inflammatory subtype), thereby signifying a homeostatic and pro-resolution function of MaR1 [10].

Inflammation and its resolution are central to the processes of vascular injury and repair, which are directly relevant to clinical problems such as failure of angioplasty, stenting, and vascular grafts in patients with advanced atherosclerotic disease. In recent studies, we characterized the anti-inflammatory and pro-resolving activities of resolvin-D1 (RvD1) in human VSMC and in a rabbit model of vascular injury induced by balloon angioplasty [7]. In the present study, we sought to investigate the biological activity of MaR1 in vascular cells, and to define a potential role for MaR1 as a therapeutic target in vascular healing.

## Materials and Methods

### Reagents, cells and treatment protocol

Human greater saphenous veins discarded at the time of coronary or peripheral bypass grafting operations at The University of California- San Francisco (approved by the Institutional Review Board; UCSF Committee on Human Research- Number: 10-03395; the committee waived the need for informed consent) were used to prepare primary cell cultures of EC and VSMC, as described previously [11]. VSMC were maintained in Dulbecco's Modified Eagle's Medium (DMEM; low glucose; HyClone, Logan, UT) containing 10% FBS (Life Technologies, Grand Island, NY) penicillin/streptomycin/amphotericin B (Lonza 1760) and used between passages 2 and 5. EC (passage 2 to 7) were maintained in Media 199 with Earle's Balanced Salt Solution (Hyclone, Logan, UT) supplemented with 10% FBS, penicillin/streptomycin/amphotericin B (Lonza 1760), ECGS (BD Cat no. 356006) and heparin (17.5 U/ml). U937 monocytes were maintained in RPMI-1640 Medium (Hyclone Laboratories, Logan, UT) supplemented with antibiotics (Lonza 1760) and 10% FBS, 1% Glutamine, 1% Sodium Pyruvate and 1.2% HEPES. MaR1 (7R, 14S-dihydroxy-4Z,8E,10E,12Z,16Z,19Z-docosahexaenoic acid) and resolvin-D1 (7S, 8R, 17S-trihydroxy-4Z,9E,11E,13Z,15E,19Z-docosahexaenoic acid) were obtained from Cayman Chemicals ((Ann Arbor, MI). In most experiments (except where indicated) we utilized a pre-treatment protocol where cells were exposed to MaR1 at indicated doses for 30 min prior to the addition of TNF-α.

### Monocyte adhesion assay

**Figure 1:**
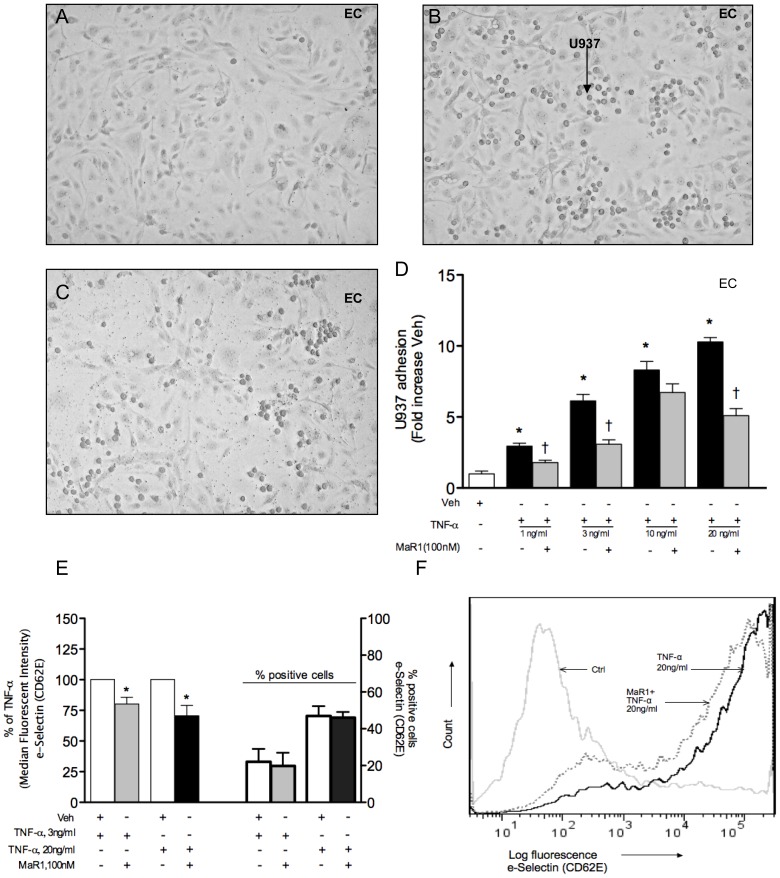
MaR1 attenuates TNF-α induced monocyte adhesion. (A, B, C) Endothelial cells grown in 24-well plates were treated with vehicle (A, B) or 100 nM MaR1 (C) for 30 min, followed by TNF-α at 1 ng/ml (B, C) for 4 hr and adhesion of labeled U937 monocytes were visualized under a microscope. (D) EC were grown in 96-well plates and adhesion of labeled U937 monocytes (4 hr post TNF-α) were quantified using a fluorescence plate reader. Treatment protocol of MaR1 was same as (Fig 1A, B, C). N≥4. N =  each well of a 96-well plate. ANOVA (oneway) with Dunnett's posthoc test (p = 0.003). *:p≤0.05 compared to vehicle control, †: p≤0.05 compared to TNF-α alone (t-test). (E) EC grown in 12-well plates were treated with vehicle or 100 nM MaR1 as (Fig 1A, B, C) and after 4 hr of TNF-α, analyzed for cell surface E-selectin expression by flow cytometry. N≥5. N =  each well of 12-well plate. *:p≤0.05 compared to TNF-α alone (t-test). Error bar  =  SEM. (F) Representative histogram of (E) for TNF-α at 20 ng/ml.

VSMC and EC, grown to 100% confluency in black 96-clear bottom plates were utilized in a static monocyte adhesion assay as described previously [12]. In brief, VSMC and EC were pre-treated with MaR1 for 30 min and activated with TNF-α for 4 hr. U937 monocytes were labeled with calcein-AM (Life Technologies, Carlsbad, CA) for 30 min at 37°C in PBS and were washed once and resuspended in PBS at a concentration of 1 million cells/ml. Labeled monocytes (0.2 million/well) were added to EC or VSMC after 4 hr of TNF-α incubation and allowed to incubate for 20 min at 37°C and 5%CO_2_. In separate experiments (Fig S1B), only the monocytes were incubated with MaR1 at various nM concentrations along with calcein-AM for 30 min at 37°C and after one wash with PBS, were placed on top of TNF-α activated VSMC (0.2 million/well). Unbound monocytes were washed off with cold PBS and fluorescence measurements (excitation 494 nm, emission 517 nm) of bound monocytes were made with a plate reader (SpectraMax M2e Molecular Devices, Sunnyvale, CA). To visualize adhesion response in EC (Fig 1 A, B, C) cells were grown in 24 well plates to 100% confluency and after performing the adhesion assay (same protocol as above), cells were washed twice with ice-cold PBS, fixed with methanol for 10 min and were visualized under a microscope with 10× objective.

### Measurement of reactive oxygen species (ROS)

For measurement of ROS in VSMC, we used the cell-permeable reagent “dihydroethidium” (DHE) as an indicator of superoxide, which binds to superoxide anions and is rapidly oxidized to ethidium bromide, which then binds to DNA and stains the nucleus red. VSMC were seeded in 8-well chamber slides (EMS, Hatfield, PA) at the concentration of 15000 cells per chamber and were treated with MaR1 the next day in serum free media for 30 min, followed by 4 hr of TNF-α (10 ng/ml). DHE was added at a final concentration of 3 µM per well and incubated for 30 min at 37°C and 5%CO_2_, protected from light. VSMC were then washed 3 times in warm PBS (in dark) and mounted with “Vectashield” mounting medium with DAPI nuclear counter-stain (Vector Laboratories, Burlingame, CA). Fluorescence was detected in a fluorescence microscope, allowing the detection of DHE at excitation/emission wavelength of 488 nm/590 nm. Multiple images (random) were taken from each treatment group (wells) and the mean nucleus-DHE fluorescence per nucleus area was calculated from all images of each well (group) using Image J software (NIH).

TNF-α is known to induce an apoptotic effect in EC and the release of cytochrome c from mitochondria into the cytoplasm is known to give a false positive signal for DHE, interfering with superoxide detection in EC [13]. Therefore in EC, we used CellRox Deep Red reagent (Life Technologies, Carlsbad, CA) as an indicator of ROS (mainly O_2_^⋅-^ and OH^⋅^ radicals). The cell-permeant dye is non-fluorescent in reduced state and binds preferentially to superoxide and hydroxyl radicals emitting a fluorescent signal with absorption/emission maxima at 644 nm/665 nm. Endothelial cells were seeded in human fibronectin coated 96-well black plates at a density of 50000 cells/well and were assayed the next day for ROS. The treatment protocols were similar to VSMC but the TNF-α dose was 1 ng/ml and we analyzed ROS production 2 hours post TNF-α. This was based on a preliminary time-course study in EC that showed a peak in the CellRox signal at 2 hr post TNF-α (data not shown).

### Western blotting

VSMC and EC were lysed in CellLytic M buffer (Sigma, Cat no. C2978) and were saved in −80°C after three pulses of low watt sonication on ice. The lysates were centrifuged at 21000 g for 20 min and the supernatants (whole cell lysates) were run on Mini-Protean TGX gels (Biorad, Hercules, CA), transferred on PVDF membranes and probed with appropriate primary antibodies. Sources of primary antibodies are as follows: NOX4 (Cat no. ab109225) from Abcam Inc (Cambridge, MA); NOX2 (Cat no. 20782), NOX1 (Cat no. SC5821) and IκB-α (Cat no. SC371) from Santa Cruz biotechnology (Dallas, TX); iNOS (PA-1036) from Thermo Scientific-Pierce (Rockford, IL); Phospho IKK α/β (Ser 176/180, Cat no. 2694) and IKK (Cat no. 2684) from Cell Signaling Inc (Danvers, MA). We used streptavidin bound Q-dot nano crystals (Life Technologies, Carlsbad, CA) for detection of the proteins bound to biotinylated secondary antibodies. For iNOS, we used HRP labeled secondary antibodies (Santa Cruz Biotechnology, Cat no. SC2030) and detected bands using Western Bright Quantum HRP substrate (Advansta, Menlo Park, CA).

### Array for analysis of secreted cytokines

Protein expression analysis of inflammatory cytokines were performed using “human antibody inflammation arrays” (Raybiotech, Norcross, GA, Cat no. AAH-INF-3) according to the manufacturer's protocol. Briefly, EC and VSMC were grown to confluency in 6-well plates and were serum starved for 24 hrs, followed by MaR1 treatment (30 min) and TNF-α for 18 hrs. Conditioned cell culture media was saved at −80°C for antibody microarray analysis. Data was normalized to protein content per well. Densitometric analysis was performed using Biorad Chemidoc Software (Image Lab 4.0.1).

### Immunofluorescence for p65 localization

Cells were seeded on 8-well chamber slides (EMS, Hatfield, PA) and after treatment, were briefly rinsed in PBS and fixed with 2% paraformaldehyde for 20 min at room temperature, followed by permeabilization in ice-cold acetone (10 min at −20°C) and 1% Triton-X100 (20 min at room temperature). Cells were incubated in a humidified chamber overnight with anti-p65 antibody (Santa Cruz Biotechnology, Dallas, TX, Cat no. SC-372) at 4°C, followed by ALEXA-Fluor 488 tagged secondary antibody (Life Technologies, Carlsbad, CA) and were visualized under a fluorescence microscope. Quantitation of fluorescent signals in nucleus and cytoplasm were performed using GIMP 2.8 software (www.gimp.org).

### cAMP assay

Cyclic AMP assay was performed using an ELISA kit (Enzo Life Sciences Inc, Farmingdale, NY, Cat no. ADI-900-066). Confluent VSMC and EC were grown in 24-well plates and following treatment, cells were lysed in 0.1 N HCl with 1% TritonX-100 and subsequently cAMP was acetylated and assayed following manufacturer's instructions. Values were calculated from a standard curve generated from acetylated-cAMP supplied by the manufacturer.

### Flow cytometry

Endothelial cells grown to 90% confluency in 12-well plates were used for flow cytometry analysis of E-selectin following a previously published protocol [14]. Since cell surface antigens are sensitive to trypsin we first stained the cells with E-selectin antibody, followed by trypsin digestion. After treatment protocol, EC in each well were washed twice with warm PBS and blocked for 10 min with mouse IgG1 in PBS (with 0.5% FBS) in an orbital shaker followed by APC-conjugated mouse-antihuman E-selectin (clone 68-5H11, BD Pharmingen, Cat# 551144) for 30 min in the dark at room temperature. EC were trypsinized briefly with trypsin/EDTA (0.05% Trypsin, Hyclone, Cat # SH30236.01) for 4 min, washed, resuspended in PBS (with 0.5% FBS) and analyzed in BD FACSVerse flow cytometer. Median fluorescence intensity (MFI) and percentage APC positive cells were analyzed by FlowJo software.

### Reporter assay

The NF-κB luciferase reporter plasmid (pGL4.32[luc2P/NF-κB-RE/Hygro] Vector) and renilla luciferase reporter plasmid (pGL4.74[h-Rluc/TK] vector) were purchased from Promega corporation (Madison, WI). The NF-κB plasmid contains 5 repeats of NF-κB response element that drives transcription of the luciferase reporter gene luc2P (*Photinus pyralis* ). The renilla pRL-TK Vector was used as an internal control reporter vector and contains the herpes simplex virus thymidine kinase (HSV-TK) promoter to provide low to moderate levels of Renilla luciferase expression in co-transfected mammalian cells. VSMC were seeded in 96-well plates at 70% confluency and were co-transfected with 75 ng of DNA (both vectors) in serum and antibiotic-free DMEM media for 1 day, followed by MaR1 or vehicle pre-treatment of 30 min and TNF-α (10 ng/ml) for 6 hr. Cells were then lysed and induced NF-κB reporter activity was corrected for the constitutive renilla luciferase expression using a Dual Luciferase Kit (Promega, WI, Cat no. E1910) and a Glomax-20/20 luminometer. Results were subtracted from background reading obtained from a non-transfected control.

## Results

### MaR1 attenuates TNF-α –induced monocyte adhesion to vascular cells

Exposure of vascular cells to pro-inflammatory mediators like TNF-α results in an increase in the adhesion of leukocytes through receptor-ligand interactions (e.g. integrins). TNF-α (1–20 ng/ml) induced a 2.9–10.3 fold increase in U937 adhesion to EC (Fig 1 D) and a 1.7–2.4 fold increase in U937 adhesion to VSMC (Fig S1A). Pre-incubation of EC and VSMC with MaR1 (100 nM) resulted in a significant attenuation of the TNF-α induced adhesion response. This decrease in adhesion was not due to cell death as measured by cytotoxicity (MTT) assays in both cell types (data not shown). We also observed (data not shown) inhibition of monocyte adhesion with MaR1 at 10 nM with TNF-α at 10 ng/ml(VSMC) and 1 ng/ml(EC). In a different experiment, we pre-treated only the monocytes with MaR1 (0.1–100 nM) and exposed them to TNF-α activated VSMC to examine direct effects of MaR1 on monocytes. We observed a dose-dependent attenuation of U937 adhesion to activated VSMC, with a maximum inhibition of 46% with 100 nM MaR1 (Fig S1B).

In order to investigate the mechanisms associated with this inhibition of cell-cell adhesion, we examined cell surface VCAM-1 and ICAM-1 in both cell types, and E-selectin expression in EC following TNF exposure (Fig 1E, F). We did not identify a significant reduction in cell surface VCAM-1 and ICAM-1 expression by MaR1 (10–100 nM, data not shown), however we observed a significant, modest decrease (20–30%) in endothelial E-selectin expression (Fig 1E) by MaR1 (100 nM).

### MaR1 abrogates TNF-α induced oxidative stress and NOX expression in vascular cells

We selected the dosage of TNF-α at 1 ng/ml for EC and 10 ng/ml for VSMC for all subsequent experiments, as these doses gave optimum induction of the experimental parameters studied.

**Figure 2:**
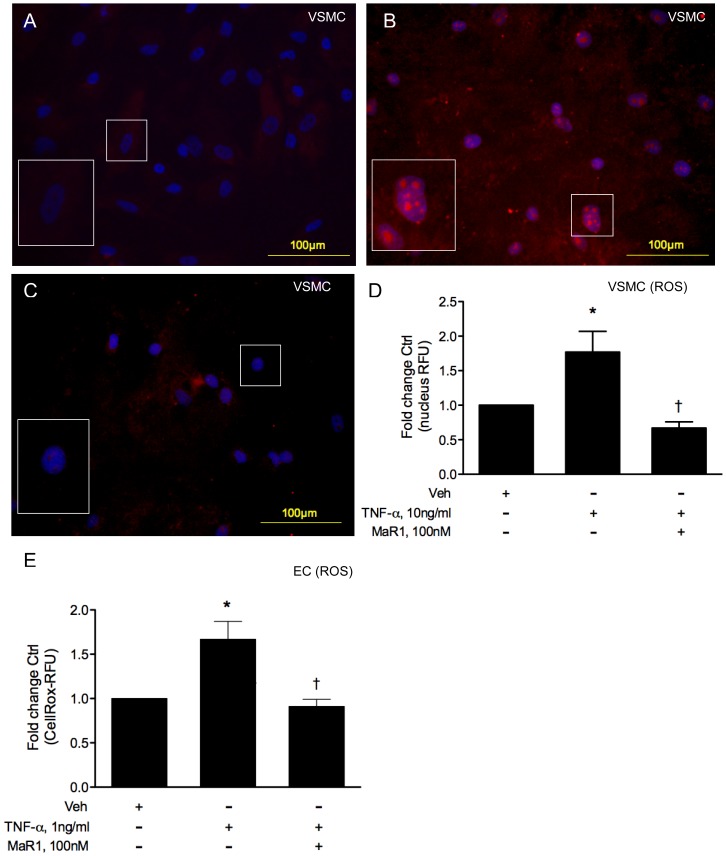
MaR1 attenuates TNF-α induced ROS production in vascular smooth muscle and endothelial cells. (A–C) Representative images of dihydroethidium/DHE stained (red) vascular smooth muscle cells counterstained with DAPI (nucleus). VSMC were grown in 8-well chamber slides and received vehicle (A, B) or 100 nM of MaR1 (C) for 30 min, followed by TNF-α, 10 ng/ml for 4 hr (B, C). (D) Quantitative analysis of mean DHE intensity (nucleus RFU/area of nucleus) of vascular smooth muscle cells, N≥3 per group. N =  each well of an 8-well chamber slide. (E) EC grown in 96-well plates, received 100 nM of MaR1, 30 min prior to TNF-α and ROS production was measured 2 hr post TNF-α, using a ROS specific dye CellRox Deep Red reagent, N≥3 per group. N =  each well of a 96-well plate. *:p≤0.05 compared to vehicle control, †: p≤0.05 compared to TNF-α alone (t-test). Error bar  =  SEM.

TNF-α binds to its receptor TNFR-I in VSMC and EC and results in ROS production in a time and dose-dependent manner. A family of ROS producing enzymes, classified as NADPH-oxidases (NOX) form the major source of TNF-α induced ROS [15], [16], [17]. We investigated the extent of ROS production under TNF-α exposure and analyzed expression of the major ROS producing NOX isoform, NOX-4 (in both VSMC and EC), NOX-1 (VSMC) and NOX-2 (EC). TNF-α (4 hr treatment) caused a significant increase in dihydroethidium (DHE) fluorescence in the nuclei of VSMC (1.77 fold Ctrl, ±0.30 SEM), indicating formation of fluorescent ethidium bromide-DNA adducts (Fig 2B, D). MaR1 treatment at 100 nM caused a significant decrease in DHE fluorescence below baseline values (0.67 fold Ctrl, ±0.09 SEM, p = 0.001) in VSMC and significantly decreased ROS generation (0.91 fold ctrl ±0.08 SEM) in EC.

**Figure 3:**
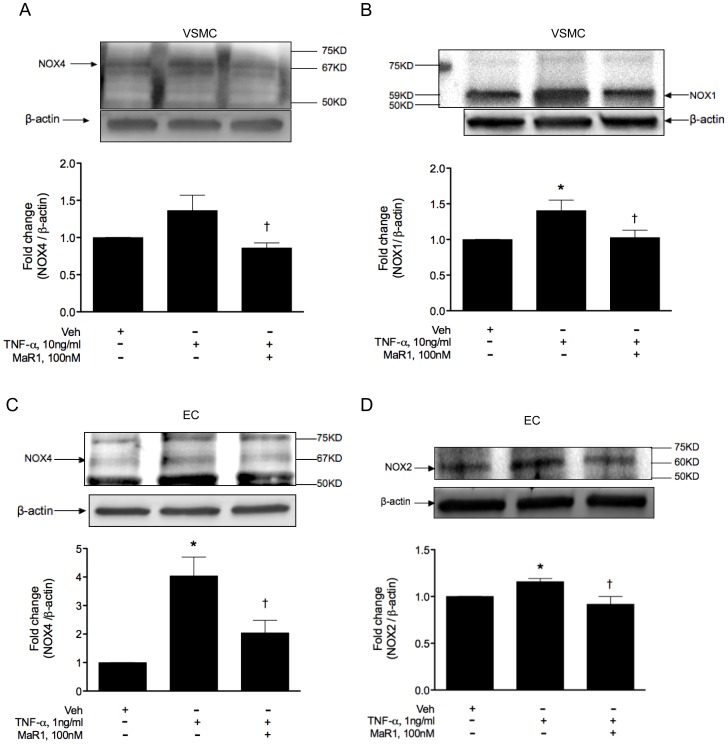
MaR1 attenuates TNF-α induced expression of NADPH-oxidases. VSMC (A, B) and EC (C, D) were treated with 100 nM of MaR1 for 30 min, followed by TNF-α for 6 hr. After TNF-α treatment, cells were lysed and probed for NOX-4, NOX-1 and NOX-2. (A) N = 14 per group, (B) N = 17 per group, (C) N≥7 per group, (D) N = 3 per group. *:p≤0.05 compared to vehicle control, †: p≤0.05 compared to TNF-α alone (t-test). N = each well of a 6-well plate. Error bar  =  SEM.

We investigated expression of NADPH-oxidases (NOX) in order to see if the reduced ROS generation was related to altered NOX expression. In VSMC, NOX4 showed up as a clearly defined band at 67 KD (isoform NOX4B) as found by others [18] whereas in EC there were a few non-specific bands in addition to the predicted 67KD weight. In this study, we have quantified this predominant NOX4 isoform (NOX4B) in both cell type. We found TNF-α to up-regulate NOX4 in VSMC (p = 0.08) 4 hr post TNF-α addition (Fig 3A), whereas MaR1 (100 nM) caused a significant attenuation of NOX-4 expression (p = 0.02 compared to TNF-α alone). In EC, MaR1 (100 nM) showed similar inhibitory effect on NOX4 expression (p = 0.042), 6 hr post TNF-α addition (Fig 3C). In VSMC, there was a significant attenuation in NOX-1 levels by 100 nM MaR1 (Fig 3B, p = 0.048) and in EC, MaR1 almost completely abolished NOX-2 expression (Fig 3D, p = 0.046).

### MaR1 attenuates TNF-α induced pro-inflammatory pathways

**Figure 4:**
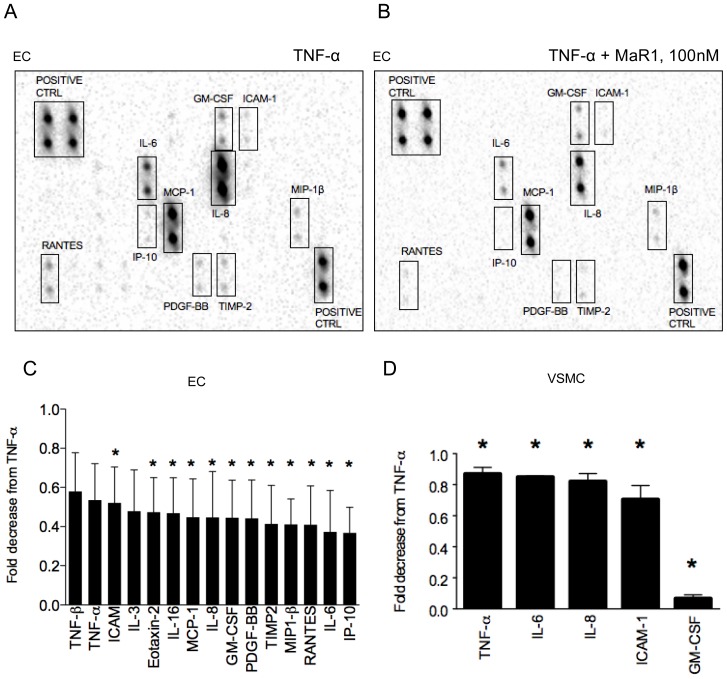
MaR1 attenuates TNF-α induced inflammatory pathways. EC were treated with 100 nM MaR1 for 30 min followed by 18 hr of TNF-α (1 ng/ml), after which the conditioned medium was analyzed for the presence of 40 different inflammatory mediators using antibody arrays. (A, B) Representative images of three independent experiments done on EC. (C) After densitometric analysis of individual spots (in duplicate) normalized to protein content (cell lysates), inflammatory mediators that were down-regulated significantly in TNF-α+ MaR1 (t-test: p≤0.05), compared to TNF-α alone are shown in the bar-graph, N = 3. (D) Graphical representation of down-regulated inflammatory proteins found in the media from VSMC that underwent same treatment protocol as (Fig 4 A–C) but with TNF-α at 10 ng/ml. N = 3. *:p≤0.05 compared to TNF-α alone (t-test). N =  each well of a 6-well plate. Error bar  =  SEM.

TNF-α augments endothelial pro-inflammatory gene transcription and subsequently the release of cytokines in the extracellular milieu that amplify local inflammation in a paracrine fashion [19], [20]. We looked at the effect of MaR1 on the levels of these secreted inflammatory mediators using an antibody-based membrane array. Out of the 40 proteins we studied using the human inflammation array-3 (refer: http://www.raybiotech.com/c-series-human-inflammation-array-3-8.html), we found many to be significantly down-regulated (∼50%–60%), 18 hr post TNF-α (1 ng/ml) by MaR1 (100 nM) in EC (Fig 4C). In VSMC, MaR1 (100 nM) caused a modest but significant decrease in several mediators (Fig 4D) with the strongest inhibitory effect seen in GM-CSF levels (Fig 4D). Additionally, in endothelial cells, we observed a dramatic attenuation of expression of inducible nitric oxide synthase (iNOS) by MaR1 (Fig S2).

**Figure 5:**
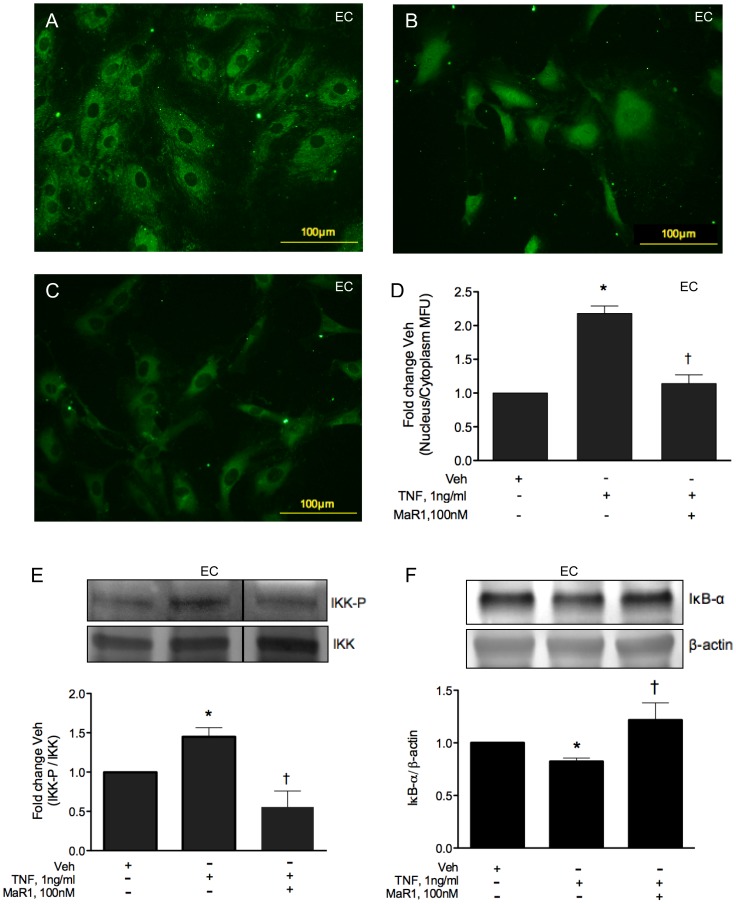
MaR1 attenuates TNF-α induced NF-κB activation in endothelial cells. (A–C) Representative images of nuclear translocation of p65 NF-κB subunit in EC treated with vehicle (A), vehicle + TNF-α, 2 hr (B), 100 nM of MaR1 (30 min) + TNF-α, 2 hr (C). (D) Quantitative analysis of p65 translocation (ratio of nuclear to cytoplasmic fluorescence) in EC. N≥3 where N =  each well of an 8-well chamber slide. (E) Endothelial whole cell extracts were analyzed for phospho- and total-IKK, 15 min post TNF-α addition that received 100 nM MaR1, 30 min prior to TNF-α. N = 3 where N =  one 10 cm plate. *:p≤0.05 compared to vehicle control, †: p≤0.05 compared to TNF-α alone (t-test). (F) EC received vehicle or 100 nM MaR1 for 30 min and TNF-α for 1 hr, after which they were lysed and probed for IκB-α. *:p≤0.05 compared to vehicle control, †: p≤0.05 compared to TNF-α alone (t-test) Error bar  =  SEM.

**Figure 6:**
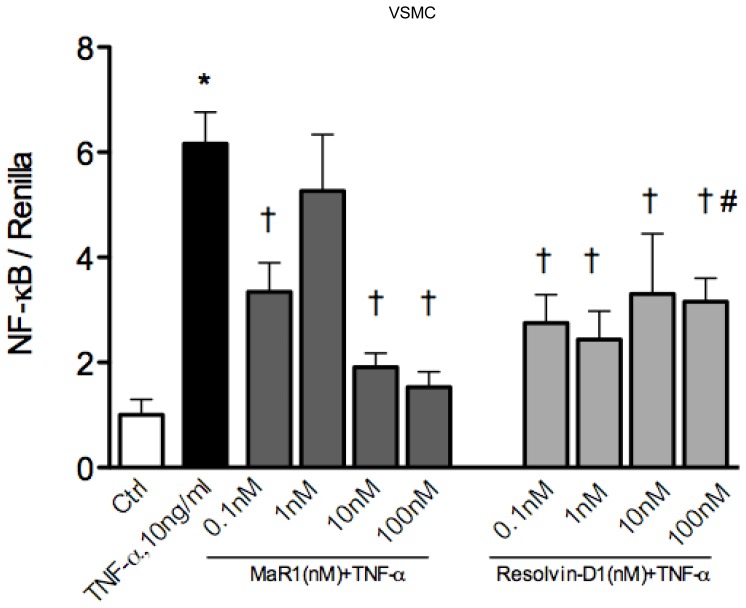
MaR1 and Resolvin-D1 attenuates TNF-α induced NF-κB reporter activity in vascular smooth muscle cells. VSMC transfected with firefly luciferase (NF-κB) and renilla luciferase vectors were treated with MaR1 and resolvin-D1, followed by TNF-α for 6 hr and were analyzed for firefly and renilla luciferase activity. Net NF-κB activity is shown as a ratio of firefly luciferase activity normalized to renilla luciferase for transfection efficiency. N≥6 per group. *:p≤0.05 compared to vehicle control, †: p≤0.05 compared to TNF-α alone (One way ANOVA with Dunnett's post hoc test). #: p = 0.01 (t-test) compared to MaR1, 100 nM +TNF-α. N =  each well of a 96-well plate. Error bar  =  SEM.

TNF-α signaling is known to involve the coordinated activity of multiple transcription factors in endothelial and smooth muscle cells (e.g. NF-κB, AP-1), resulting in enhanced transcription of a spectrum of inflammatory mediators [21]. The NF-κB family of transcription factors (p65/p50 heterodimer being the most abundant type) are involved with activation of various pro-inflammatory genes in response to TNF-α [22]. Binding of TNF-α to its receptor TNFR-I results in activation of a series of down-stream signaling intermediates that phosphorylate I-kappa kinases (IKK) which in turn phosphorylates I-kappa α and results in its proteosomal degradation, releasing p65 from the I-kappa α/p65-p50 complex and its translocation into the nucleus [23]. We examined these key steps in VSMC and EC exposed to TNF-α with or without MaR1 preteatment at 100 nM. In both EC (Fig 5) and VSMC (Fig S3), MaR1 significantly reduced p65 nuclear translocation in response to TNF-α (Fig 5C, D; Fig S3 C, D). In endothelial cells, TNF-α enhanced IKK-phospho levels to 1.45 fold control and MaR1 decreased the phosphorylation to 0.55 fold control levels (Fig 5E). In smooth muscle cells TNF-α caused a 31% increase in IKK-p whereas MaR1 brought down IKK-phospho levels below control levels (Fig S3E). As shown in Fig 5F and Fig S3F, there was a significant inhibition of IκB-α degradation in both cell types by MaR1(100 nM). Finally, we looked at the net extent of NF-κB activation over a longer time-period (6 hrs) in VSMC, utilizing a luciferase based reporter assay (Fig 6). We tested MaR1 and the pro-resolving mediator resolvin-D1 at four different doses in order to compare dose-dependent effects of these two distinct lipid mediators. Our results show a significant inhibition of NF-κB activation by both MaR1 and Resolvin-D1, however at 100 nM, MaR1 was slightly more effective that resolvin-D1 (Fig 6).

### MaR1 stimulates cAMP production in naive and TNF-α activated VSMC and EC

**Figure 7:**
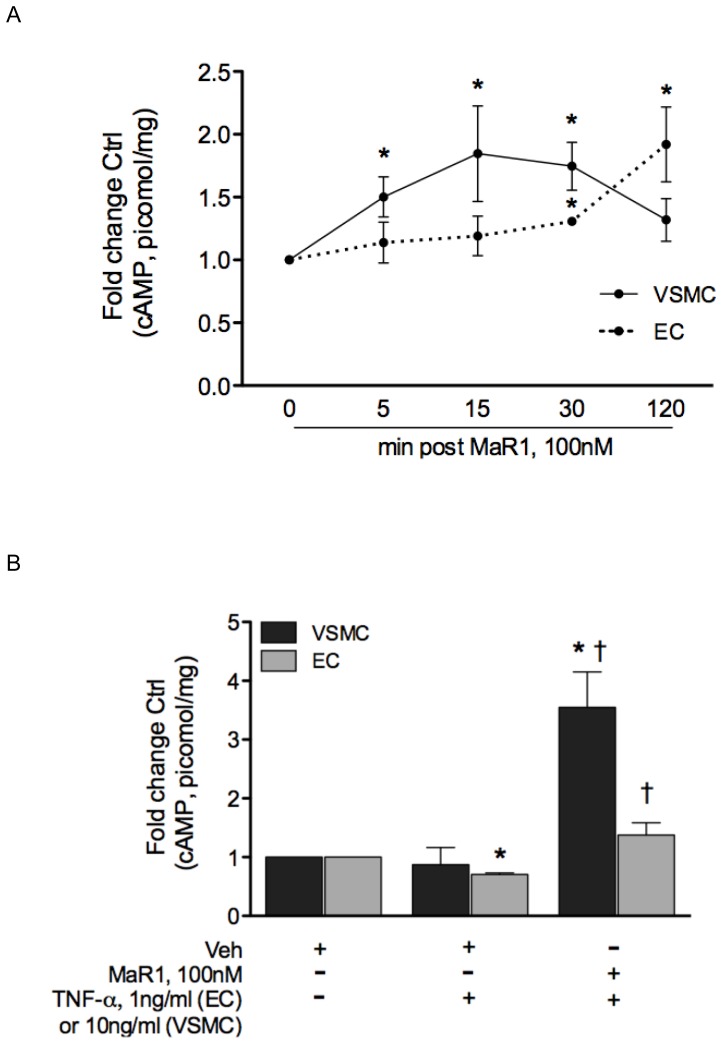
MaR1 increases cAMP levels in vascular smooth muscle and endothelial cells. (A) VSMC and EC were seeded to confluency in 24-well plates and were treated with 100 nM MaR1 for the indicated time-points and cAMP levels were determined and normalized to mg protein. N = 3 per time-point (B) VSMC and EC were treated with vehicle or MaR1 (100 nM) for 30 min, followed by TNF-α (10 ng/ml for VSMC, 1 ng/ml for EC) for 120 min and assayed for cAMP. N = 4. *:p≤0.05 compared to vehicle control (0 min) (t-test); †: p≤0.05 compared to TNF-α + vehicle (t-test). N =  each well of a 24-well plate. Error bar  =  SEM.

Since cyclic-AMP modulates inflammatory signals in a wide variety of cells, and is involved with promoting an anti-inflammatory [24] and anti-oxidant effect in vascular cells [25], we determined the effects of MaR1 on intracellular cAMP levels in both naive and TNF-α activated VSMC and EC. There was a time-dependent increase in intracellular cAMP levels in both cell types by MaR1, where vascular smooth muscle cells showed enhanced elevation of cAMP in comparison to endothelial cells in early time-points (Fig 7A). There was a 50%, 84% and 75% increase in cAMP in VSMC, 5 min, 15 min and 30 min post addition of MaR1 and a modest 30% increase in cAMP levels in EC, 30 min post MaR1 treatment. We investigated cAMP levels, 2 hr post TNF-α in both cell types (Fig 7B) where VSMC/EC received 100 nM MaR1 or vehicle for 30 min followed by TNF-α for 2 hrs. We found that in both cell types MaR1 significantly elevated cAMP levels in TNF-α treated cells. In VSMC, cAMP levels were 3.5 fold (±0.60 SEM) higher in MaR1 treated group versus vehicle control group, compared to 0.87 fold (±0.28 SEM) in TNF-α alone group (Fig 7B). In EC, TNF-α exposure led to reduced cAMP levels to 0.70 fold control (±0.02 SEM, p≤0.05) whereas MaR1 pre-treatment caused an increase in cAMP levels of 1.37 fold (±0.20 SEM) control (Fig 7B).

## Discussion

Acute vascular injury (e.g. resulting from angioplasty, stents, bypass grafting etc.) involves endothelial and smooth muscle cell activation resulting in adhesion and sequestration of monocytes and neutrophils and subsequent tissue injury to the vessel wall. The inflammation that succeeds the vascular injury induces a phenotypic switch in VSMC that results in their proliferation and migration to the *tunica intima*, resulting in development of intimal hyperplasia. Therefore, modulation of the inflammatory response forms one of the pre-eminent therapeutic strategies to attenuate the severity of vascular injury. The findings reported in this study have clinical relevance to a broad range of vascular diseases e.g. atherosclerosis, vascular injury associated restenosis, diabetes and peripheral vascular disease among many others as most of them have persistent vascular inflammation as an important contributor in the pathogenesis of the disease. Since the discovery of pro-resolving lipid mediators by Serhan et al. [1] in an acute murine model of peritonitis, there has been an exponential growth in the number of investigations on DHA and EPA derived SPMs, especially in relation to inflammatory diseases. Resolvin-E1 is under active investigation in clinical trials for dry eye, asthma and other diseases (Source: www.clinicaltrials.gov). Compared to other SPMs, relatively little is known about the biological activity of the macrophage synthesized lipid mediator MaR1. Moreover, its effects on endothelial and smooth muscle cells have not been characterized. Since Maresins and D-series resolvins share the same precursor (DHA), and we have previously shown that D-series resolvins exert potent anti-inflammatory and homeostatic effects in vascular cells [7], [26] we hypothesized MaR1 to possess similar properties.

We have observed an anti-adhesive effect of MaR1 on both cell types. When MaR1 was incubated with monocytes alone, we also saw an inhibition of the adhesive effect, which might imply that MaR1 interferes with monocyte function. Flow-cytometry based analyses showed that MaR1 causes a 20–30% reduction in cell surface E-selectin expression, which is involved with “rolling” phenomenon of monocytes that precedes firm adhesion. Surprisingly, we did not see any significant inhibition of cell surface expression of VCAM-1 and ICAM-1 in EC and VSMC by MaR1. Investigations are underway in our lab in order to better characterize the mechanisms of reduced adhesion in the presence of MaR1, looking at multiple pathways that may affect monocyte interactions to EC and VSMC.

TNF-α causes generation of reactive oxygen species in endothelial and smooth muscle cells primarily through activation of NADPH-oxidase 4 (NOX-4). Other cell-specific isoforms of NOX, like NOX-1 (in VSMC) and NOX-2 (in EC) are also involved with TNF-α-induced ROS generation but to a much lesser extent compared to NOX-4 [17], [27]. Two recent studies show RvD1 [28] and RvE1 [29] to reduce ROS production in macrophages, however to our knowledge, no study has examined the effects of MaR1 on ROS generation in EC or VSMC. Our results show MaR1 to attenuate ROS production, associated with reduced NOX protein expression in both cell types. As ROS is known to play a potentially detrimental role in inflammation, the observed beneficial effects of MaR1 could be partially linked to attenuation of the ROS response. Additional studies involving mRNA and protein expression of components of NOX-4 enzyme complex (e.g. p22 phox), NOX-4 enzyme activity, and characterization of ROS species (hydrogen peroxide, superoxide etc.) are under way to further elucidate mechanisms of ROS attenuation.

TNF-α activates multiple pro-inflammatory transcription factors in EC and VSMC that result in gene transcription and release of the mediators (e.g. cytokines and chemokines) in the extracellular milieu, that act in a paracrine and autocrine fashion to modulate the inflammatory responses. We investigated the effects of MaR1 on extracellular release of 40 different inflammatory mediators in TNF-α activated EC and VSMC 18 hr post TNF-α, and found MaR1 to attenuate a number of the mediators including chemokines and chemoattractants like Interferon gamma induced protein-10 (IP-10), MCP-1, RANTES, MIP-1β, IL-8, Eotaxin-2, IL-16 and cytokines such as GM-CSF and IL-3 which are involved with proliferation and maturation of cells of myeloid lineage. Interestingly, MaR1 also attenuated PDGF-BB release from endothelial cells, an important regulator of VSMC proliferation and migration. MaR1 has been shown *in vivo* to block NF-κB activation in colonic tissues in a murine colitis model [9], however MaR1 was found to have no inhibitory effect on NF-κB activation in human bronchial epithelial cells [8]. Our results show a strong inhibitory effect of MaR1 on NF-κB activation in both cell types, involving several key steps involved with NF-κB activation including phosphorylation of IKK and IκB-α degradation.

RvD1 and RvE1 receptors have been identified, and their actions are known to be mediated through GPCRs in a pertussis toxin sensitive fashion, indicating involvement of Go/Gi type GPCRs [7], [30], [31], [32], [33]. MaR1 receptors are still unknown, however a recent report showed partial inhibition of the effects of MaR1 in dorsal root ganglion neurons, in the presence of pertussis toxin, suggestive of a decrease in cAMP mediating MaR1's effects in neurons [3]. In this study, we report MaR1 to elevate intracellular cAMP levels in naive smooth muscle and endothelial cells. A recent study also showed time-dependent elevation of cAMP and PKA activity by resolvin-D1 in mouse RAW 264.7 macrophages [28]. Moreover, we found that MaR1 led to increased cAMP levels in TNF-α treated cells. Cyclic-AMP has been shown to impart anti-inflammatory actions on cytokine activated human endothelial cells and vascular smooth muscle cells, by blocking NF-κB activation [34], [35] and reducing adhesion molecule expression [24], [36], [37], [38], and therefore forms an important line of investigation related to MaR1's anti-inflammatory actions on vascular cells.

Increased levels of 14-HpDHA have been found in subcutaneous fat surrounding foot wounds in patients with peripheral vascular disease (PVD), suggesting activation of resolution pathways involving MaR1 in PVD [39]. In another study, bacterial lipopolysaccharide was found to enhance the synthesis of 14-HpDHA and MaR1 in human Caco-2 epithelial cells and foam macrophages [40]. These observations highlight the importance of MaR1 in activating “anti-inflammatory” and “resolution” signaling pathways in the inflammatory zone or region and underscores its importance in turning “ON” pro-resolution pathways in these inflammatory diseases. In summary, in the present study we report MaR1 to impart a strong anti-inflammatory phenotype in human vascular smooth muscle cells and endothelial cells, associated with reduced monocyte adhesion and TNF-α induced production of ROS and inflammatory mediators. At the molecular level, we found MaR1 to reduce NOX expression and inhibit NF-κB activation and increase intracellular cAMP levels in both cell types. Hence, we conclude that MaR1 has potent anti-inflammatory actions in vascular cells of human origin and modulates signaling pathways under basal and TNFα-stimulated conditions. These findings suggest a therapeutic potential for maresins and their emergence as a novel family of DHA-derived SPMs to treat vascular inflammatory diseases.

## Supporting Information

Figure S1 **MaR1 attenuates TNF-α induced monocyte (U937) adhesion in VSMC.** (A) U937 monocyte adhesion in TNF-α activated VSMC that received 30 min pre-treatment of vehicle or MaR1(100 nM). (B) U937 monocyte adhesion on TNF-α activated (10 ng/ml) VSMC where only the monocytes were pre-treated with MaR1 (0.1–100 nM) for 30 min. N≥4 per group. ANOVA (oneway) with Dunnett's posthoc test (Fig S1A: p = 0.007, Fig S1B:p = 0.003). *:p≤0.05 compared to vehicle control, †: p≤0.05 compared to TNF-α alone. N =  each well of a 96-well plate. Error bar = SEM.
(TIF)

Figure S2 **MaR1 attenuates TNF-α induced iNOS expression in endothelial cells.** Confluent EC grown in 6-well plates were treated with vehicle or TNF-α (1 ng/ml) for 6 hrs with/without 30 min pretreatment with 100 nM MaR1. Whole cell lysates were probed with iNOS and beta-actin. N = 3.*:p≤0.05 compared to vehicle control, †: p≤0.05 compared to TNF-α alone (t-test). N =  each well of a 6-well plate. Error bar  =  SEM.
(TIF)

Figure S3 **Effect of MaR1 on TNF-α induced NF-κB activation in VSMC.** VSMC were treated with vehicle (A, B) or 100 nM MaR1 (C) for 30 min followed by TNF-α (10 ng/ml, B, C) for 2 hr. (A–C) Representative images of VSMC showing nuclear translocation of p65 (in green) (D) Quantitative analysis of A, B, C. N≥3. N =  each chamber of an 8-well chamber slide. (E, F) VSMC whole cell extracts were analyzed for phospho- and total-IKK (15 min post TNF-α) and IκB-α (1 hr post TNF-α). (E) N = 3. N =  each10 cm plate and (F) N = 5. N =  each well of a 6-well plate. Graph represents densitometric analyses of western blots. *:p≤0.05 compared to vehicle control, †: p≤0.05 compared to TNF-α alone. Error bar  =  SEM.
(TIF)
