# Supplementary figures and images for: The Pro-Resolving Lipid Mediator Maresin 1 (MaR1) Attenuates Inflammatory Signaling Pathways in Vascular Smooth Muscle and Endothelial Cells
Source: PLoS One. 2014 Nov 19;9(11):e113480. doi: 10.1371/journal.pone.0113480 (PMC4237455; doi:10.1371/journal.pone.0113480)

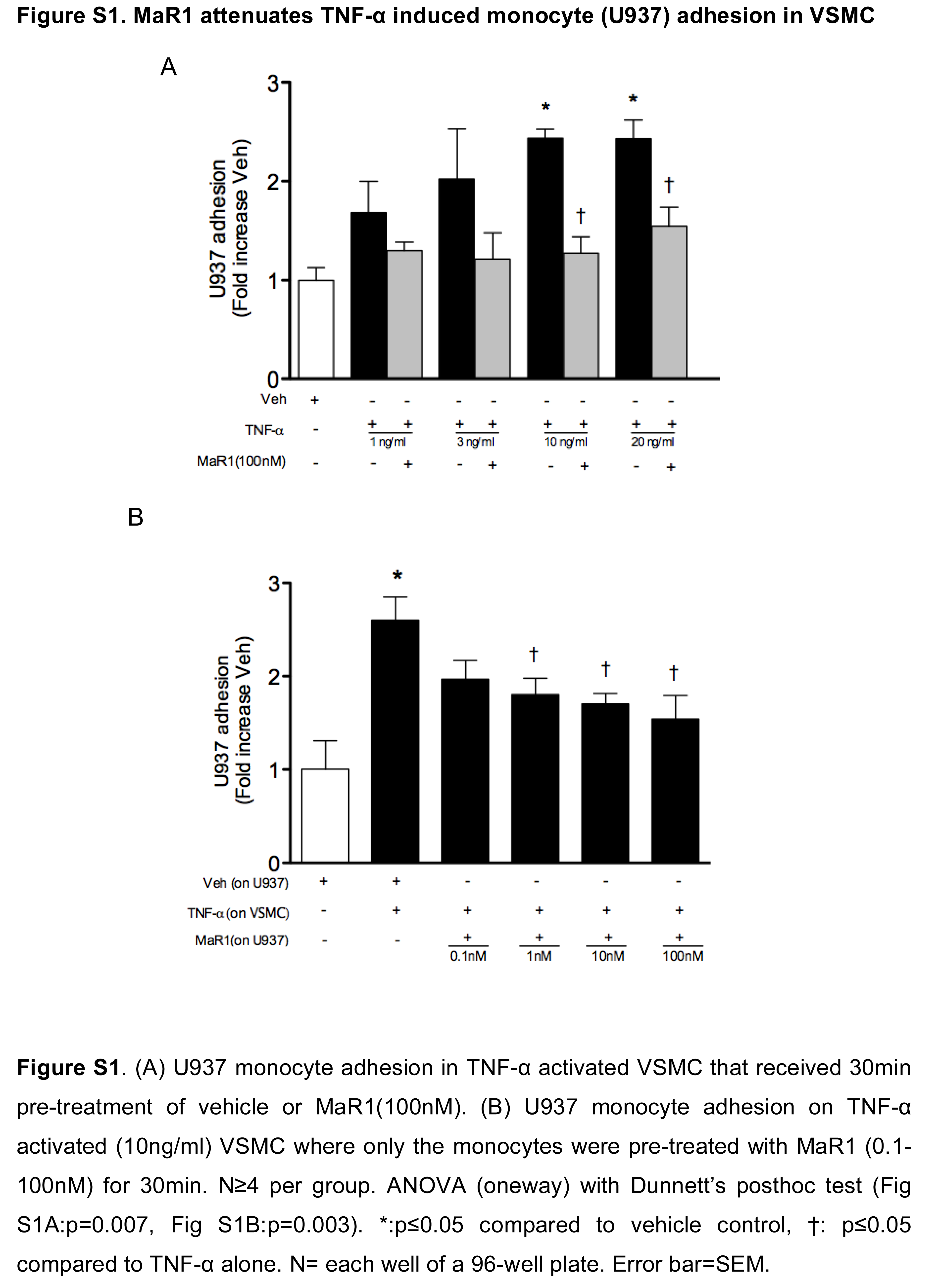

Supplement: Figure S1 — MaR1 attenuates TNF-α induced monocyte (U937) adhesion in VSMC. (A) U937 monocyte adhesion in TNF-α activated VSMC that received 30 min pre-treatment of vehicle or MaR1(100 nM). (B) U937 monocyte adhesion on TNF-α activated (10 ng/ml) VSMC where only the monocytes were pre-treated with MaR1 (0.1–100 nM) for 30 min. N≥4 per group. ANOVA (oneway) with Dunnett's posthoc test (Fig S1A: p = 0.007, Fig S1B:p = 0.003). *:p≤0.05 compared to vehicle control, †: p≤0.05 compared to TNF-α alone. N = each well of a 96-well plate. Error bar = SEM. (TIF) [file pone.0113480.s001.tif]

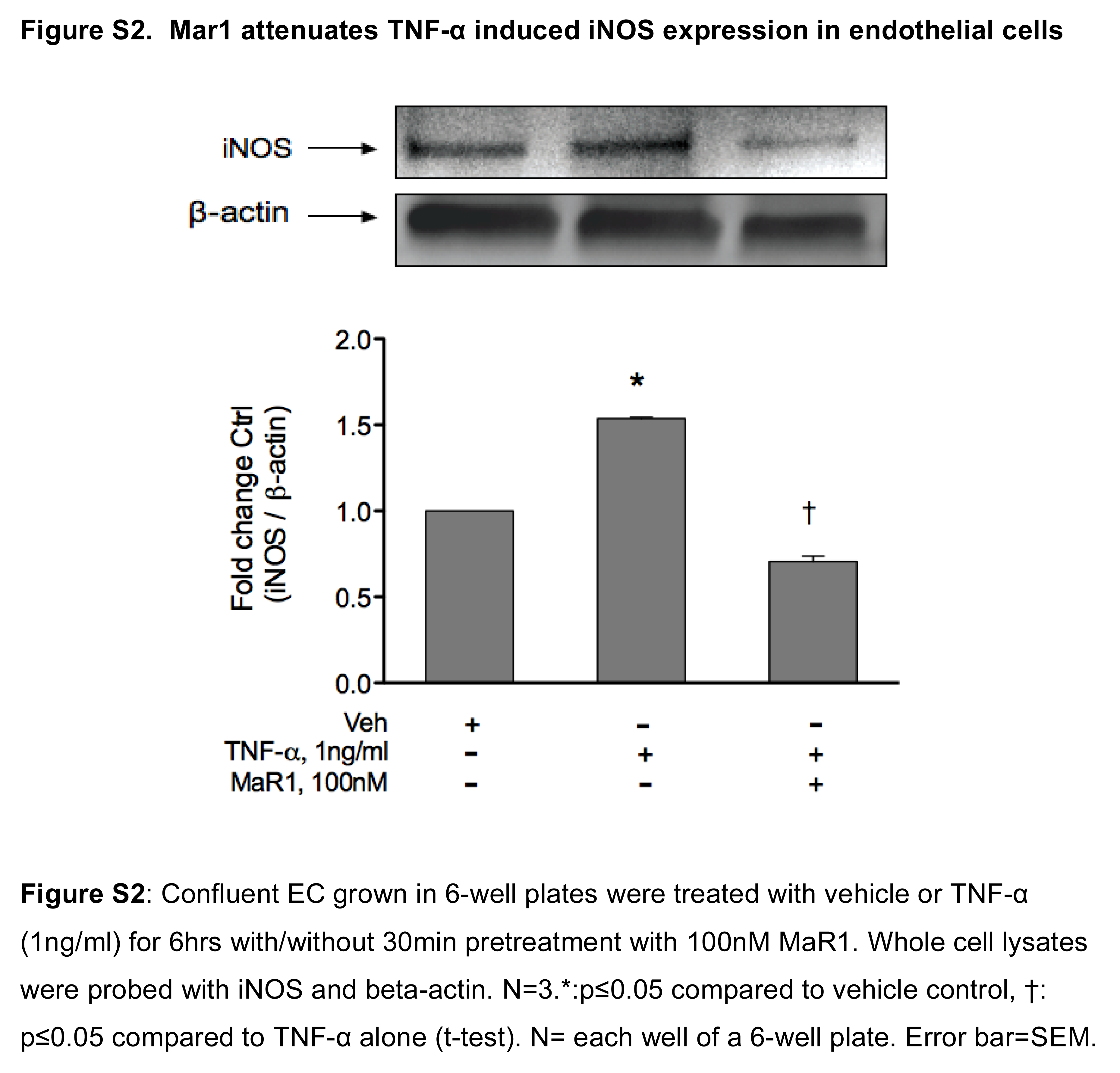

Supplement: Figure S2 — MaR1 attenuates TNF-α induced iNOS expression in endothelial cells. Confluent EC grown in 6-well plates were treated with vehicle or TNF-α (1 ng/ml) for 6 hrs with/without 30 min pretreatment with 100 nM MaR1. Whole cell lysates were probed with iNOS and beta-actin. N = 3.*:p≤0.05 compared to vehicle control, †: p≤0.05 compared to TNF-α alone (t-test). N = each well of a 6-well plate. Error bar = SEM. (TIF) [file pone.0113480.s002.tif]

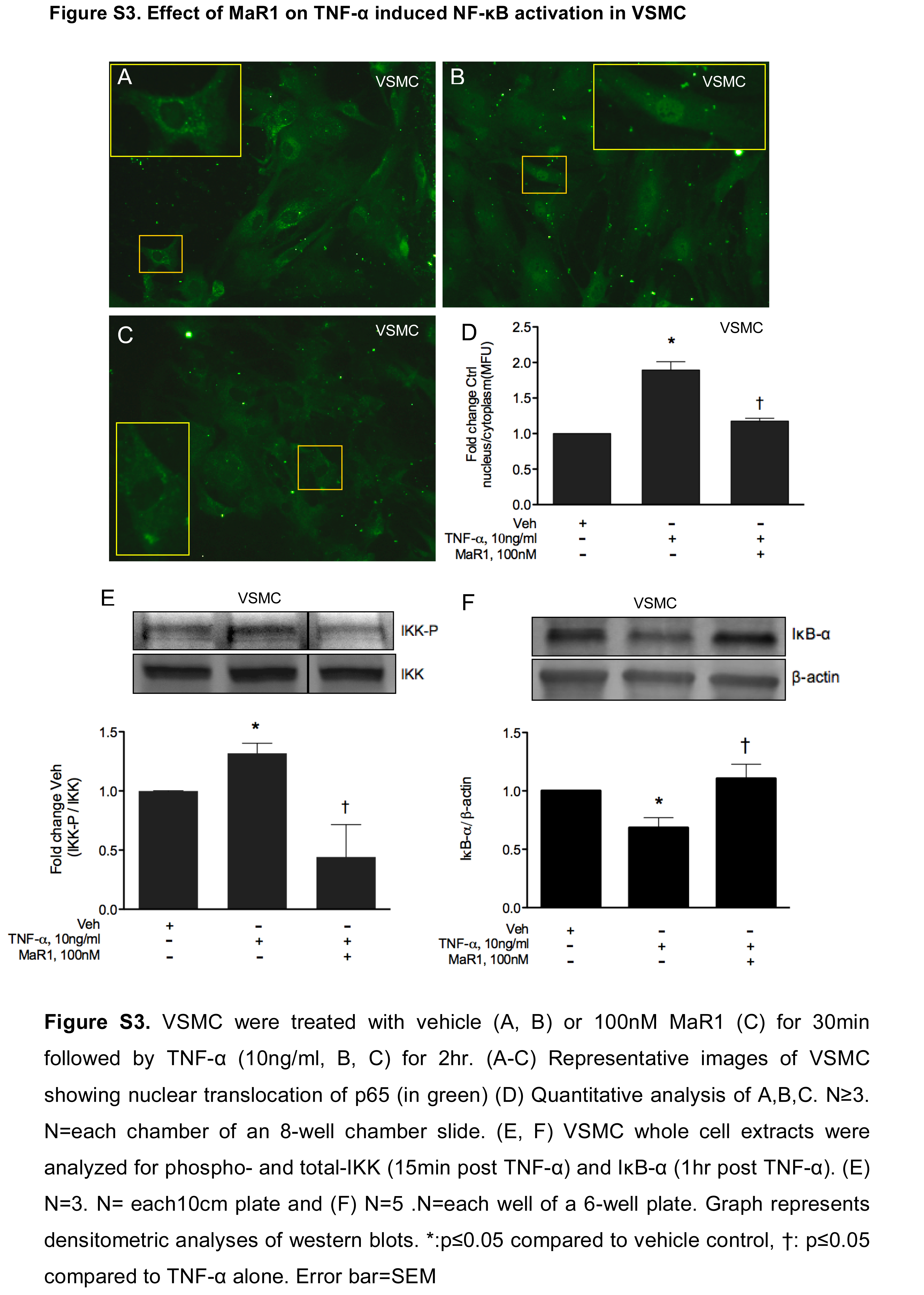

Supplement: Figure S3 — Effect of MaR1 on TNF-α induced NF-κB activation in VSMC. VSMC were treated with vehicle (A, B) or 100 nM MaR1 (C) for 30 min followed by TNF-α (10 ng/ml, B, C) for 2 hr. (A–C) Representative images of VSMC showing nuclear translocation of p65 (in green) (D) Quantitative analysis of A, B, C. N≥3. N = each chamber of an 8-well chamber slide. (E, F) VSMC whole cell extracts were analyzed for phospho- and total-IKK (15 min post TNF-α) and IκB-α (1 hr post TNF-α). (E) N = 3. N = each10 cm plate and (F) N = 5. N = each well of a 6-well plate. Graph represents densitometric analyses of western blots. *:p≤0.05 compared to vehicle control, †: p≤0.05 compared to TNF-α alone. Error bar = SEM. (TIF) [file pone.0113480.s003.tif]
